# Liquid biopsy to identify biomarkers for immunotherapy in hepatocellular carcinoma

**DOI:** 10.1186/s40364-021-00348-y

**Published:** 2021-12-20

**Authors:** Huang Ao, Zhang Xin, Zhou Jian

**Affiliations:** 1 Department of Liver Surgery and Transplantation, Zhongshan Hospital, Fudan University; Key Laboratory of Carcinogenesis and Cancer Invasion (Fudan University), Ministry of Education; Shanghai Key Laboratory of Organ Transplantation, Zhongshan Hospital, Fudan University, Shanghai, 200032 China; 2 Institute of Biomedical Sciences, Fudan University, Shanghai, 200032 China; 3 State Key Laboratory of Genetic Engineering, Fudan University, Shanghai, 200032 China; 4 Liver Cancer Institute, Zhongshan Hospital, Fudan University, 136 Yi Xue Yuan Road, Shanghai, 200032 China

**Keywords:** Liquid biopsy, Immunotherapy, Immune checkpoint inhibitors, Hepatocellular carcinoma, ctDNA, CTC

## Abstract

The past years have witnessed the vigorous development of immunotherapy, mainly immune checkpoint inhibitors (ICIs) targeting the programmed cell death-1 (PD-1) protein and its ligand, PD-L1, and cytotoxic T-lymphocyte-associated antigen-4 (CTLA-4). Indeed, ICIs have largely revolutionized the management and improved the prognosis of patients with intermediate and advanced hepatocellular carcinoma (HCC). However, biomarker-based stratification of HCC patients for optimal response to ICI treatment is still of unmet need and again, there exists the necessity to dynamically monitor treatment effect in real-time manner. The role of conventional biomarkers in immunotherapy surveillance is largely limited by spatial and temporal tumor heterogeneity whereas liquid biopsy seems to be promising to circumvent tumor heterogeneity to identify candidate patients who may response to immunotherapy, to dynamically monitor treatment effect and to unveil resistance mechanism. Herein, we provide a thorough review about the potential utility of liquid biopsy in immunotherapy for HCC and discuss its future perspectives.

## Background

During the past three years, the management of hepatocellular carcinoma (HCC) has been drastically improved with the application of immunotherapy, especially after the advent of immune checkpoint inhibitors (ICIs), namely antibodies targeting the programmed cell death receptor-1 (PD-1), the anti-programmed death 1 ligand (PD-L1), the cytotoxic T-lymphocyte antigen 4 (anti-CTLA-4), and most recently, the LAG-3 (Lymphocyte-activation-gene-3). To date, several ICIs and the combination of ICIs, or ICIs with anti-angiogenic antibody have been approved for the treatment of HCC, either as first-line or second-line agents [1, 2]. Although phase III trials of ICI monotherapies in HCC have been negative, such failure has merely influenced the use of ICIs in clinical practice and trials evaluating the combination therapies of ICIs, ICIs with molecular targeted agents or other modalities are still worth expectation [3]. Moreover, evaluation of ICIs as neoadjuvant treatment may provide novel insights and decrease tumor recurrence and metastasis after hepatectomy (Table 1).

**Table 1:** Ongoing clinical trials evaluating neoadjuvant immunotherapy in HCC

Identifier No.	Interventions	Setting	Candidates	Primary outcome
NCT04930315	Camrelizumab + apatinib	Phase 2, open label	BCLC B/C, or CNLC IIa-IIIb; technically resectable	1-year tumor recurrence-free rate
NCT03630640	Nivolumab	Phase 2, open label	BCLC A; receiving electroporation	2-year RFS
NCT04727307 (AB-LATE02)	Atezolizumab	Phase 2, open label	Percutaneous Radiofrequency	2-year RFS
NCT04615143	Atezolizumab;	Phase 2, single arm	Resectable recurrent HCC	Pathological response rate
NCT04658147	Nivolumab With or Without Relatlimab	Phase 1	Potentially Resectable HCC	No. of patients who complete treatment and surgery
NCT03916627	Cemiplimab	Phase 2	Resectable HCC	Significant tumor necrosis

Despite that ICIs have resulted in significant therapeutic benefit and prolongation of survival in the whole, the extent of benefit in ICI is not uniform. Not all patients benefit from immunotherapy and most patients would eventually experience disease progression. Although immunotherapies are largely thought to have fewer adverse effects than chemotherapy and molecular targeted therapy, the immune-related adverse events (irAEs) such as myocarditis and thyroiditis could be lethal and it’s thus crucial to identify patients who are at risk of therapy related toxicity. Moreover, hyperprogressive disease (HPD) after treatment with ICIs could make the condition much worse. Thus, predictive biomarkers of ICI response are urgently needed to guide treatment decision and patient selection, as well as to better understand and overcome mechanisms of resistance.

### Conventional biomarkers for immunotherapy in HCC

Till now, conventional biomarkers for cancer immunotherapy fall into the following categories: the expression of PD-L1, specific genetic signatures/somatic mutations, tumor mutation burden (TMB), dMMR/MSI (deficient in mismatch repair /microsatellite instability), tumor microenvironment (TME), and gut microbiome. Intuitively, PD-L1 is the ideal biomarker for cancer immunotherapy. Gao et al. reported for the first time PD-L1 expression status in HCC and provided the rationale of immunotherapy targeting the PD-1/PD-L1 pathway [4]. Other researchers also reported a positive correlation between PD-L1 expression and poor survival in HCC, which further support the usage of ICIs in HCC patients falling within this category [5–7]. Moreover, high expression of PD-L1 in the adjacent liver tissue predicted shorter overall survival (OS) and disease-free survival (DFS), meaning the expression of PD-L1 in the peritumoral liver tissue could also be an indicator for adjuvant immunotherapy after radical hepatectomy [8]. However, the approval of nivolumab from CHECKMATE-040 and pembrolizumab from KEYNOTE-224 in the second-line setting for advanced HCC were independent of PD-L1 or immune cell expression [9, 10]. Moreover, the expression of PD-L1 in HCC was not all consistently high [11], limiting its practical utility as biomarker for immunotherapy in HCC.

Interestingly, for HCC patients who receive liver transplantation (LT) and experience recurrence, expression of PD-L1 is a solid contraindication for ICI treatment [12]. Recurrence of HCC after LT are largely caused by the immunosuppressive microenvironment brought by immunosuppressants and ICI might thus provide survival benefit in these patients. Although cases have been reported that patients with recurrent, disseminated HCC after LT were successfully treated with ICIs [13, 14], one should note that introduce of ICIs in these LT recipients inevitably improves immunity and may result in organ rejection and liver failure. Thus, only patients with negative PD-L1 staining should be prescribed ICI to treat tumor recurrence following LT.

TMB has been found highly correlated with the activity of anti–PD-1 therapies across multiple cancers [15] and it has been approved by the United States Food and Drug Administration (FDA) as a criterion for pembrolizumab treatment across different tumor types. In resectable lung cancer, TMB was predictive of the pathological response in patients with neoadjuvant PD-1 blockade [16]. Next generation sequencing (NGS) based genomic profiling work has reported several high TMB associated genetic signatures which may probably promote antitumor immunity and serve as biomarker for ICIs in HCC [17].

Partially like that with TMB, the cancer genome with dMMR contains exceptionally high numbers of mutation-associated neoantigens and may might be recognized by the immune system. For instance, colorectal cancers (CRC) with dMMR had high immunogenicity and were sensitive to immune checkpoint blockade with PD-1 antibodies [18, 19] whereas misdiagnosis of MSI or dMMR status in metastatic CRC displaying MSI or dMMR would lead to primary resistance ICIs [20]. In patients with resectable primary gastric cancer, MSI-H could robustly predict immunotherapy response and correlate with longer DFS and OS [21]. Similarly, a recent meta-analysis of randomized controlled trials including the phase III KEYNOTE-062, CheckMate-649, JAVELIN Gastric 100 and KEYNOTE-061 trials, also suggested MSI-H as a positive predictive factor to immunotherapy in patients with advanced gastric cancer [22]. In critically ill patients with end-stage cancers, MSI-H status was proposed to be used in clinical practice as a tumor-agnostic predictive biomarker to select candidate for salvage immunotherapy [23]. Indeed, the importance of dMMR/MSI-H status has been further validated in multiple solid tumors and proved to be highly responsive to immunotherapy across different tumors of origin and FDA had already granted an accelerated approval to the anti PD-1 antibody pembrolizumab for adult and pediatric patients with agnostic unresectable/metastatic dMMR or MSI-H cancers [24].

Literally, TME and gut microbiome would surely play an important role in immunotherapy, especially for HCC. Through transcriptome-based immunogram analysis, immunological microenvironment including Wnt/β-catenin activation, high combined positive score of PD-L1, and increased infiltration of CD8+ cells in HCC were found significantly associated with longer survival in HCC patients receiving ICI treatments [25]. By analyzing the expression profile of immune-modulating genes in HCC, Cao et al. found 4 immune subtypes of HCCs with different survivals: patients with B7-H3^low^/CD8^high^ or CD47^low^/CD8^high^ had the best while those with B7-H3^high^/CD8^low^ or CD47^high^/CD8^low^ have the worst survival. Such immune classification system may facilitate select HCC patient who would benefit from ICI treatment [26]. HCCs with a discrete population of PD1-high CD8+ T cells might benefit from combined immune checkpoint blockade-based therapies [27]. NASH (non-alcoholic steatohepatitis) -related aberrant T cell activation causing tissue damage which leads to impaired immune surveillance may be the reason that non-viral HCC, particularly NASH-HCC, might be less responsive to immunotherapy [28].

Gut microbiome, which is critical for the development and regulation of innate and adaptive immunity, may also influence treatment outcomes of ICIs [29, 30]. The gut-liver axis suggests a close connection between liver and gut, whit increasing evidence has proposed a key role of microbiome in the development of HCC [31–34]. Gut microbiome may thus be particularly meaningful in the immunotherapy for HCC. In fact, significant differences on the diversity and composition of gut microbiota in HCC who received ICIs have been found between responders and non-responders [35]. Dynamic variation of the gut microbiome characteristics may be predictive of the efficacy of immunotherapy, contributing to disease monitoring and decision making [36]. Though no association of gut microbiome with the ICI treatment efficacy in patients with HCC has also been reported, there still remained enriched microbiome subclones in patients with disease control [37].

### Tumor heterogeneity challenges the effect of existed biomarkers in immunotherapy

Till now, few biomarkers have been successfully translated into clinical implication of HCC. One possible reason for such biomarker directed immunotherapy is tumor heterogeneity. Tumor heterogeneity is the key feature of carcinogenesis and is linked with medication therapy failure [38, 39]. HCC is a typical tumor of extreme heterogeneity at genomic [40], epigenetic [41], transcriptional [42], and protein [43] level. Single-cell analysis also demonstrated that HCC cancer stem cells are composed of phenotypically, functionally, and transcriptomically heterogeneous subpopulations [44]. Even so, the tumor heterogeneity of HCC could still be classified into the following four categories: interpatient heterogeneity indicates differences between patients of the same tumor histology, intertumoral heterogeneity means differences between tumor foci in the same patient, intratumoral heterogeneity (ITH, spatial heterogeneity) is the differences among cells of the same tumor foci, and temporal heterogeneity describes the temporal changes in a tumor along treatment.

The four types of heterogeneity collectively contribute to medication therapy failure. Interpatient heterogeneity implies that a simple biomarker may not fit the whole patient population and personalized ICI treatment modality is necessary for better treatment response. However, the co-existence of intra- and inter-tumoral heterogeneity, with the latter one being much more profound and multifocal tumors being common in HCC, challenges precision immunotherapy since single tumor biopsy evaluation of PD-L1 expression or profiling genomic information could not represent the landscape of the whole tumor, let alone for multifocal HCCs. Thus, primary treatment resistance may happen. Even for tumors who are sensitive initially, the temporal heterogeneity, which is caused by selective pressure during treatment, would result in the expansion of rare subclones of resistant tumor cells and deem the eventual failure of agents. Indeed, single-cell transcriptomic analysis of liver tumors from patients treated with immunotherapy found an increase in tumor cell state heterogeneity was tightly linked to treatment response and patient prognosis [45].

### Liquid biopsy, the promising biomarker for cancer immunotherapy

In the past years, liquid biopsy has emerged as an appealing source of new biomarkers and has been widely used in the management of cancer including HCC [46]. Accordingly, studies evaluating the predictors of benefit from ICI treatment have displayed a burgeoning interest in liquid biopsy. The analysts consist of liquid biopsy include circulating tumor DNA (ctDNA), circulating tumor cells (CTC), lymphocyte subpopulations, exosomes and metabolites to name a few. Liquid biopsy had several advantages over conventional single-site tissue sampling or serum protein markers, including higher sensitivity and specificity, non-nonvasiveness, dynamic monitoring, and the most important of all, overcoming the limit of spatial and temporal heterogeneity [47]. For HCC, tissue access is not always easily available and a single biopsy could hardly represent the whole genetic information, let alone in the case of multiple and huge HCCs. In the process of treatment (TACE, targeted therapy and immunotherapy), the selection pressure resulted clonal evolution surely leads to tumor genome change and drug resistance, and finally treatment failure. However, the genetic information from tissue biopsy prior to treatment does not contribute to treatment decision Meanwhile, repeated tumor biopsy is invasive and not operable in clinical practice. In such circumstance, liquid biopsy could be highly useful since venipuncture is nearly non-invasive and the genetic information from ctDNA and CTC has been demonstrated representative of the tumor genome [48]. Thus, with the application of liquid biopsy, it’s able to reveal a much more comprehensive tumor genome and dynamically monitor the genetic change along treatment [49]. In general, liquid biopsy could help select candidate HCC patients who would benefit from immunotherapy in several aspects.

### Implementation of personalized immunotherapy

Ideally, assessment of PD-L1 expression in tumor tissues could predict ICI response; however, there is a growing interest in assessment of PD-L1 mRNA and protein levels in circulating extracellular vesicles, which may have the potential to predict response to anti-PD-1/PD-L1 antibodies [50, 51]. For instance, CTCs from metastatic prostate cancer patients heterogeneously express immune checkpoints including B7-H3, PD-L1, PD-L2, and CTLA-4, which suggests the detection of CTCs with these immune checkpoints may select patients suitable for immunotherapy [52]. In patients with metastatic genitourinary cancer received immunotherapy, higher CTC counts at baseline and presence of specific CTC morphologic subtypes, PD-L1^+^ CTCs, are associated with shorter survival [53]. Similarly, in head and neck squamous cell carcinoma patients, CTCs with PD-L1 overexpression are prognostic and the absence of PD-L1 overexpression in CTCs at the end of treatment strongly correlated with complete response [54]. Detection of PD-L1 positive CTC in peripheral blood of HCC patients is feasible and lays the foundation for real-time surveillance and individualized immunotherapy [55].

On the other hand, NGS based ctDNA detection in HCC patients may be of extreme usefulness for precision immunotherapy. ctDNA carries plenty of genetic information derived from tumor tissues and it could serve as supplement to tumor biopsy or solely provide profiling of tumor genome with high coverage [56]. Not only TMB but also genetic signatures and MSI/dMMR could all be analyzed through ctDNA profiling. In the Phase I trial of small case series bearing solid tumors, TMB calculated from ctDNA correlated with that form tumor tissues and decrease in mutation variant allele frequency during treatment was observed in responders [57]. By retrospective analysis, Gandara et al. found ctDNA TMB reproducibly identified NSCLC (non-small cell lung cancer) patients who had obtained clinically significant improvements in PFS from atezolizumab as second-line treatment or higher [58]. Recently, commercial ctDNA platform for quantifying bTMB from plasma samples has been demonstrated to be feasible, accurate, and reproducible, with the optimal ctDNA TMB cutoff of ≥20 mut/Mb predictive of clinical benefit with durvalumab + tremelimumab versus chemotherapy [59]. More importantly, if assayed with panels containing a greater number of mutations or larger panels, ctDNA could more accurately reflect tissue TMB [60, 61] and higher TMB in ctDNA was associated with longer PFS in NSCLC patients treated by ICIs [62].

Tumor mutations profiled through cfDNA sequencing is another effective biomarker to predict immunotherapy outcomes [63]. C*TNNB1* gene mutations are reported to be predictive of response to immunotherapy in HCC patients [64, 65] but intratumoral heterogeneity and the presence of multifocal tumors challenge comprehensive molecular profiling from a single biopsy. Besides, mutational profiling of tumor biopsies taken prior to immunotherapy do not ensure continuous treatment response during the whole disease course. However, ctDNA could provide additional information of tumor mutations which were not apparent in single tumor biopsy and combining analysis of ctDNA and tumor tissue increased the detection rate of *CTNNB1* mutation [66]. More commonly, ctDNA has been demonstrated quantifiable across all HCC stages and could specially detect mutations in the key driver genes [67].

NGS has been approved for detection of MSI/dMMR in patients with metastatic CRC before ICI treatment and such method was reported to perform at least as efficiently as the reference method [68]. Combination of NGS with liquid biopsy to identify cancer patients with MSI-H is feasible in clinical practice and may overcome the limitation and invasiveness of tumor tissue biopsy [69]. A recent report has demonstrated NGS based liquid biopsy for MSI evaluation in clinical practice: 2 metastatic castration-resistant prostate cancer patients who failed multiple lines of therapy and were detected MSI-H in ctDNA assay had shown an excellent clinical response to pembrolizumab [70]. It’s thus reasonable to propose NGS of ctDNA or single cell sequencing of CTC for MSI/dMMR analsysis in HCC.

### Candidate selection for adjuvant immunotherapy

The 5-year recurrence rate of HCC after hepatectomy could be as high as 70% and adjuvant treatments to prevent recurrence after curative liver resection is of unmet need [71]. Adjuvant immunotherapy has displayed survival benefit for patients undergone radical surgery in melanoma [72], esophageal or gastroesophageal junction cancer [73]. It’s thus reasonable to propose such usage in HCC, especially in patients with high recurrence risks. In fact, immunotherapy may play pivotal role in this kind of clinical setting [74]: adjuvant immunotherapy with activated autologous cytokine-induced killer cells increased recurrence-free and overall survival in patients who underwent curative treatment for HCC [75]. Although clinical trials evaluating the effect of adjuvant immunotherapy after hepatectomy are still ongoing (Table 2), it’s not uncommon that surgical oncologist have already prescribed ICIs for patients at high risk of recurrence.

**Table 2:** Postoperative adjuvant immunotherapy trials for HCC

Identifier No.	Interventions	Setting	Primary outcome
NCT03859128 (JUPITER 04)	Toripalimab or placebo	Phase 2, 3	RFS
NCT03383458 (CheckMate 9DX)	Nivolumab or placebo	Phase 3	RFS
NCT03867084 (KEYNOTE-937)	Pembrolizumab	Phase 3, Double-blinded, Placebo controled	RFS, OS
NCT04639180	Camrelizumab plus Apatinib		
NCT04102098 (IMbrave050)	Atezolizumab plus Bevacizumab	Phase 3, open label, two arms (versus active surveillence)	RFS
NCT03847428 (EMERALD-2)	Durvalumab plus bevacizumab or durvalumab monotherapy or placebo	Phase 3, randomized, double-blind, placebo-controlled	RFS
NCT04418401 (CISLD-8)	Donafenib plus any anti-PD-1 antibody	Phase 1	1-year RFS
NCT04233840	Nivolumab and Ropeginterferon alfa-2b	Phase I/II Open Label	RFS

The underlying mechanism for indication of adjuvant immunotherapy may rest in minimal residual disease (MRD), which implies disseminated tumor cells (DTC) from the primary lesion to distant organs without any clinical or radiological evidence of metastasis at the time of initial treatment or residual tumor cells left behind after curative-intent surgery that eventually lead to local recurrence. The existence of MRD highly predicts tumor recurrence and metastasis. Moreover, DTCs could take advantage of the expression of checkpoint ligands such as PD-L1 to escape TIL antitumor activity, which means ICI-based anticancer strategy may be especially relevant against MRD. Thus, identification of HCC patients with MRD facilitates precise adjuvant immunotherapy whereas liquid biopsy has displayed extremely sensitivity for detection of MRD [76]. For example, in localized colon cancer, postoperative plasma ctDNA detected MRD and identified patients at high risk of relapse, preceding radiological recurrence with a median lead time of 11.5 months [77].

Actually, liquid biopsy guided adjuvant immunotherapy has already proved survival benefit in cancer patients. In the phase III IMvigor010 study, adjuvant atezolizumab did not prolong DFS in unselected patients with urothelial cancer who underwent surgery; however, in those who were tested positive for ctDNA, improvement in both DFS and overall survival was observed [78]. In comparison, no such differences were noted among trial participants who were negative for ctDNA in their blood in the overall study population [79].

In HCC, microvascular invasion (MVI) implies disseminated tumor cells in the adjacent liver tissues and is a common type of MRD. Interestingly, positive ctDNA status after liver resection correlated with MVI and predicted early tumor recurrence of HCC [80]. Similarly, CTC loads decreased immediately after hepatectomy in HCC patients and those with persistently high postoperative CTC load had a high probability of tumor recurrence [81]. Moreover, patients with postoperative CTC load ≥3 bear higher risk of extrahepatic metastasis after curative surgical resection of HCC and they should be recommended with close surveillance program to timely implement interventions [82]. In fact, postoperative CTC count was found to be an effective indicator for the administration of adjuvant TACE (transcatheter arterial chemoembolization) since adjuvant TACE could reduce early recurrence in HCC patients with positive CTC status after curative-intent hepatectomy [83]. Thus, detection postoperative ctDNA status or CTC load may be a viable option for HCC patients after surgery and positive ctDNA expression or CTC load could be an indication for adjuvant immunotherapy. More commonly, identifying MRD-positive HCC patients will surely provide additional benefit, regardless of the type of adjuvant ICI treatment, or even other modalities.

### Dynamic treatment surveillance

Apart from being as an indicator of adjuvant immunotherapy, liquid biopsy could also dynamically, and is especially suitable for, monitor ICI treatment response [84–87] (Table 3). Traditionally, treatment response is mainly judged by comparison of tumor size on imaging. But this kind of assessment bears the disadvantages of low sensitivity, radioactivity, and most of all, not timely enough. Besides, the treatment effects of immunotherapy are challenging to interpret since tumors often shrink slowly or become enlarged due to inflammation (radiological pseudoprogression). What’s more important, the selective pressure during immunotherapy could induce clonal evolution, which mean the tumor genome differs from that profiled before treatment. Unfortunately, conventional techniques are almost impossible to dynamically monitor such changes via longitudinal and real-time analysis.

**Table 3:** Clinical trials evaluating biomarkers of response in HCC patients treated by immunotherapy

Identifier No.	Biomarker type	Treatment	Patients
NCT05044676	Circulating Immune Cells	Atezolizumab/Bevacizumab	Advanced HCC
NCT04965454 (ExTRACT-HCC)	Genomic liquid biopsy based biomarkers	ICIs	Inoperable HCC
NCT03895970	Not specified	Lenvatinib plus Pembrolizumab	Advanced hepatobiliary tumors
NCT04642664 (ACABC)	Not specified	Apatinib plus Camrelizumab	Hepatobiliary Neoplasm
NCT03595813 (IMMUNO-SUP)	Plasma immunosuppressive actors	ICIs	Locally advanced or metastatic solid tumor
NCT03514368 (MINER)	Immunological biomarkers from blood samples	ICIs, alone or in combination	Any advanced solid tumor

Liquid biopsy, however, has the potential of determining tumor evolution patterns and therapeutic responses in a dynamic and non-invasive manner. In lung cancer and urothelial cancer, change in variant allele frequency of somatic mutations in ctDNA strongly correlated with ICIs treatment duration, activity, and outcomes [88]. Both CTC numbers and PD-L1 expression on CTCs potentially indicate disease progression during the disease course of urothelial carcinoma patients receiving ICIs [89]. Decreased ctDNA level served as an early marker of therapeutic efficacy and predicted significantly prolonged survival in lung cancer patients treated with ICIs [90]. Longitudinal analysis of ctDNA with custom NGS gene panel allowed for disease burden monitoring during immunotherapy in melanoma patients [91] and a persistently elevated ctDNA during treatment indicated poor prognosis and the necessity of combination and sequencing of subsequent therapies [92]. Moreover, undetectable ctDNA at baseline or detectable ctDNA at baseline followed by > 10-fold decrease during immunotherapy was associated with pseudoprogression while patients experienced true progression had detectable ctDNA at baseline which remained stable or increased [93]. These experience could be readily used for reference in HCC. In fact, ctDNA profiling during lenvatinib treatment was a useful marker of disease progression [94] and serial profiling of ctDNA using targeted ultra-deep sequencing correlated with systemic treatment response [95].

### Unraveling immunotherapy resistance mechanism

Nonetheless, the response to ICIs in HCC is 20–30% and patients would invariably undergo disease progression. Though other therapeutics could still be used following ICI exposure, it’s necessary to explore the mechanisms of ICI resistance and the combination of TKIs with ICI or as subsequent therapy. As mentioned above, tumor heterogeneity is the key factor contributing to immunotherapy resistance, either primary or acquired. Thus, techniques which could circumvent the obstacles brought by tumor heterogeneity can surely help unveil the underlying mechanisms and evidence from other malignancies highlights the importance of liquid biopsy in these aspects. By liquid biopsy monitoring of tumor subclones over treatment, Stein at al found ctDNA showed rapid clearance in the majority of patients mirroring a high rate of early tumor shrinkage and loss of PD-L1 on the tumor cell surface by *PD-L1* mutations abrogated direct antitumor effects of PD-L1 antibody, avelumab [96]. Moreover, such mutations were present already months before clinical resistance occurred, which supported the notion that temporal heterogeneity caused by selective pressure resulted in acquired resistance. Detection of PD-L1+ aneuploid CTCs and circulating tumor endothelial cells in histopathologic PD-L1 negative NSCLC patients indicated that ITH resulted in primary resistance to anti-PD-1 treatment [97].

Liquid biopsy could also discriminate immunotherapy resistance from pseudo-progression. Pseudo-progression happens in the early stage of immunotherapy when larger tumor lesions or new lesions occur as a result of the inflammatory reaction induced by ICI, before the tumor actually shrinks. Generally, it’s not easy to distinguish pseudo-progression from immunotherapy resistance using imaging modalities. However, the two different biological process could be effectively judged by liquid biopsy methods since in cases with pseudo-progression, the ctDNA levels decrease rapidly and persistently, whereas treatment resistance associated disease progression usually have significant increases in ctDNA amount. Indeed, Takai et al. reported a melanoma patient experienced immediate decrease of ctDNA content surge after a surge at the time of pseudo-progression [98].

## Conclusions

The advent of ICIs has greatly changed the management and outcome of HCC while biomarkers predicting ICI treatment efficacy are still of unmet need. Future studies are urgently needed to evaluate the value of utilizing liquid biopsy analytes for therapeutic decision making by identification of HCC patients who would benefit from or are nonresponders to ICI treatment. The findings on the liquid biopsy would undoubtfully reveal the fundamental role of such analytes in HCC immunotherapy, with foreseeable and not-far-to-come clinical applications.

## Data Availability

Not applicable.

## Abbreviations

HCC: hepatocellular carcinoma
ICI: immune checkpoint inhibitor
ctDNA: circulating tumor DNA
CTC: circulating tumor cell
PD-1: programmed cell death-1
PD-L1: programmed cell death-1ligand
CTLA-4: cytotoxic T-lymphocyte-associated antigen-4
irAE: immune-related adverse event
OS: overall survival
DFS: disease-free survival
TMB: tumor mutation burden
HPD: hyperprogressive disease
ITH: intratumoral heterogeneity
dMMR/MSI: deficient in mismatch repair /microsatellite instability
LT: liver transplantation
TME: tumor microenvironment
TACE: transcatheter arterial chemoembolization
NASH: non-alcoholic steatohepatitis
MVI: microvascular invasion
MRD: minimal residual disease
DTC: disseminated tumor cell
NGS: next generation sequencing
NSCLC: non-small cell lung cancer
